# *Paratype*: a genotyping tool for *Salmonella* Paratyphi A reveals its global genomic diversity

**DOI:** 10.1038/s41467-022-35587-6

**Published:** 2022-12-23

**Authors:** Arif M. Tanmoy, Yogesh Hooda, Mohammad S. I. Sajib, Kesia E. da Silva, Junaid Iqbal, Farah N. Qamar, Stephen P. Luby, Gordon Dougan, Zoe A. Dyson, Stephen Baker, Denise O. Garrett, Jason R. Andrews, Samir K. Saha, Senjuti Saha

**Affiliations:** 1 Child Health Research Foundation, Dhaka, Bangladesh; 2 Department of Medical Microbiology and Infectious Diseases, Erasmus University Medical Center, Rotterdam, the Netherlands; 3 MRC-Laboratory Molecular Biology, Cambridge, UK; 4 Institute of Biodiversity, Animal Health and Comparative Medicine, University of Glasgow, Glasgow, UK; 5 Division of Infectious Diseases and Geographic Medicine, Stanford University School of Medicine, Stanford, CA USA; 6 Department of Paediatrics and Child Health, Aga Khan University, Karachi, Pakistan; 7 Wellcome Trust Sanger Institute, Hinxton, Cambridge, CB10 1SA UK; 8 Cambridge Institute of Therapeutic Immunology and Infectious Disease, Department of Medicine, University of Cambridge, Cambridge, UK; 9 Department of Infection Biology, London School of Hygiene and Tropical Medicine, London, UK; 10 Department of Infectious Diseases, Central Clinical School, Monash University, Melbourne, VIC 3004 Australia; 11 Applied Epidemiology Team, Sabin Vaccine Institute, Washington, DC USA; 12 Department of Microbiology, Bangladesh Shishu Hospital and Institute, Dhaka, Bangladesh

**Keywords:** Bacterial genomics, Epidemiology, Bacterial infection, Pathogens

## Abstract

*Salmonella* Paratyphi A, the primary etiology of paratyphoid, is estimated to cause 3.4 million infections annually, worldwide. With rising antimicrobial resistance and no licensed vaccines, genomic surveillance is key to track and monitor transmission, but there is currently no reliable genotyping framework for this pathogen. Here, we sequence 817 isolates from South Asia and add 562 publicly available genomes to build a global database representing 37 countries, covering 1917–2019. We develop a single nucleotide polymorphism-based genotyping scheme, *Paratype*, that segregates *Salmonella* Paratyphi A population into three primary and nine secondary clades, and 18 genotypes. Each genotype is assigned a unique allele definition located on an essential gene. Using *Paratype*, we identify spatiotemporal genomic variation and antimicrobial resistance markers. We release *Paratype* as an open-access tool that can use raw read files from both Illumina and Nanopore platforms, and thus can assist surveillance studies tracking *Salmonella* Paratyphi A across the globe.

## Abstract (web summary)

The bacterium *Salmonella* Paratyphi A causes paratyphoid fever. Here, the authors sequence over 800 isolates from South Asia, build a global database representing 37 countries, and develop a genotyping tool that identifies genomic variation and antimicrobial resistance markers for surveillance studies.

## Introduction

Paratyphoid fever, caused by *Salmonella enterica* subspecies *enterica* serovar Paratyphi A (*Salmonella* Paratyphi A) is a systemic febrile illness that affects an estimated 3.4 million people each year, and causes 19,100 deaths globally^1^. The disease is clinically indistinguishable from typhoid fever, caused by *Salmonella enterica* subspecies *enterica* serovar Typhi (*Salmonella* Typhi). Much like typhoid, paratyphoid fever is also endemic in many low- and middle-income countries of South Asia and Sub-Saharan Africa, due to fecal contamination of water, food, and the environment. However, barring a few countries (e.g., China, Myanmar), paratyphoid fever is usually less prevalent than typhoid fever^2,3^. *Salmonella* Paratyphi A continues to be an inadequately studied pathogen^4^ hampering the implementation of evidence-based policies for the treatment and prevention of paratyphoid fever.

Relative to *Salmonella* Typhi, little genomic information is available on population structure, antimicrobial resistance (AMR), and spatiotemporal distribution of *Salmonella* Paratyphi A. The first *Salmonella* Paratyphi A genome was published in 2004 and has a size of 4.5 Mb, with ~4200 genes. To determine the global diversity of *Salmonella* Paratyphi A isolates, Bayesian analysis was conducted on a set of 149 *Salmonella* Paratyphi A genomes, which identified that the last common ancestor of all *Salmonella* Paratyphi A existed for at least 450 years prior to differentiating into at least seven distinct lineages (A to G) which have circulated globally^5^. Whole genome sequencing was also used to characterize clonal paratyphoid outbreaks in Cambodia^6^ and China^7^ and further extend the lineage scheme to include sub-lineages within lineage A and C. However, very few studies have characterized isolates from countries in South Asia, which contributes over 80% of all paratyphoid infections^8,9^. Available studies are sporadic, and either focused on genomes from a specific geographical location or provide no information on antimicrobial resistance markers, potential vaccine targets, and other virulence factors.

Here, we perform whole-genome sequencing of 817 *Salmonella* Paratyphi A isolates collected from Bangladesh (*n* = 528), Nepal (*n* = 156), and Pakistan (*n* = 133) and combine them with whole-genome sequence data of another 562 isolates reported in the literature to build a global database of 1379 *Salmonella* Paratyphi A isolates. To track the evolution of *Salmonella* Paratyphi A over a century, we use the existing lineage scheme and find that certain lineage and sub-lineages were not homologous, and many isolates could not be assigned a specific lineage. This motivates us to develop a single nucleotide polymorphism (SNP) based genotyping scheme, called *Paratype*. The scheme is phylogenetically informative and successfully segregates the global population structure into three primary, seven secondary, and 18 distinct subclades/genotypes. We also identify the specific antimicrobial resistance genes, mutations, and plasmids present in *Salmonella* Paratyphi A genomes and correlate these with the different genotypes.

## Results

### Whole-genome sequencing and compilation of global *Salmonella* Paratyphi A genomes

A total of 817 *Salmonella* Paratyphi A isolates were sequenced from Bangladesh, Nepal and Pakistan. The Child Health Research Foundation (CHRF) has been conducting typhoid and paratyphoid fever surveillance in Bangladesh since 1999 and has generated a biobank of 1123 *Salmonella* Paratyphi A isolates from 1999–2018^10–12^. We selected 528 of these isolates, covering years of isolation, gender, collection sites, and hospitalization status (hospitalized/out-patient), and performed whole-genome sequencing on these isolates (Supplementary Table 1). Of these 528, 180 *Salmonella* Paratyphi A isolates were collected as part of the Surveillance of Enteric Fever in Asia Project (SEAP, 2016−2019) study^13^. The SEAP study was also conducted in Nepal and Pakistan; 156 isolates were sequenced from Nepal, and 133 from Pakistan.

To contextualize these genomes, we conducted a literature search to compile all publicly available *Salmonella* Paratyphi A genomes (for which raw reads were available) to build a database of 560 additional isolates from 10 studies (Supplementary Table 2). Two reference isolates (ATCC 9150 (https://www.ncbi.nlm.nih.gov/assembly/GCF_000011885.1) and AKU_12601 (https://www.ncbi.nlm.nih.gov/assembly/GCA_006518435.1)) with complete genomes were also included. The largest dataset consisted of 254 isolates, published by Public Health England as part of their *Salmonella* surveillance^8,14^; 164 of these isolates were linked to travel, most commonly to South Asia. In our study, we assigned these isolates to the countries where the patient acquired the infection. Our final data, including the genomes we sequenced, consisted of a total of 1,379 isolates from 37 different countries, spanning over 103 years − 1917 to 2019. Most of the isolates (1112/1379; 81%) were from countries in South Asia (541 from Bangladesh, 268 from Nepal, 187 from Pakistan, and 115 from India). South Asian countries also bear a disproportionately high burden of paratyphoid fever; of the estimated 3.4 million global paratyphoid infections in 2019, 2.8 (82%) million are estimated to have occurred in South Asia^1^.

Following assembly from raw reads, the pan-genome analysis identified 6983 genes, of which 4114 genes (59% of all genes) were conserved in more than 95% of the isolates and 2335 genes (33% of all genes) were conserved in 100% of the isolates (Supplementary Fig. 1). The average genome size was 4.5 Mb with ~4300 genes, and the pan-genome does not appear to be closed (decay parameter, alpha = 0.67). Overall, 2550 genes were found to be present in less than 15% of isolates, and these included genes often found in prophages and other mobile regions, and genes encoding adhesins, antimicrobial resistance markers, and hypothetical proteins.

### Genotyping scheme for *Salmonella* Paratyphi A

**Fig. 1:**
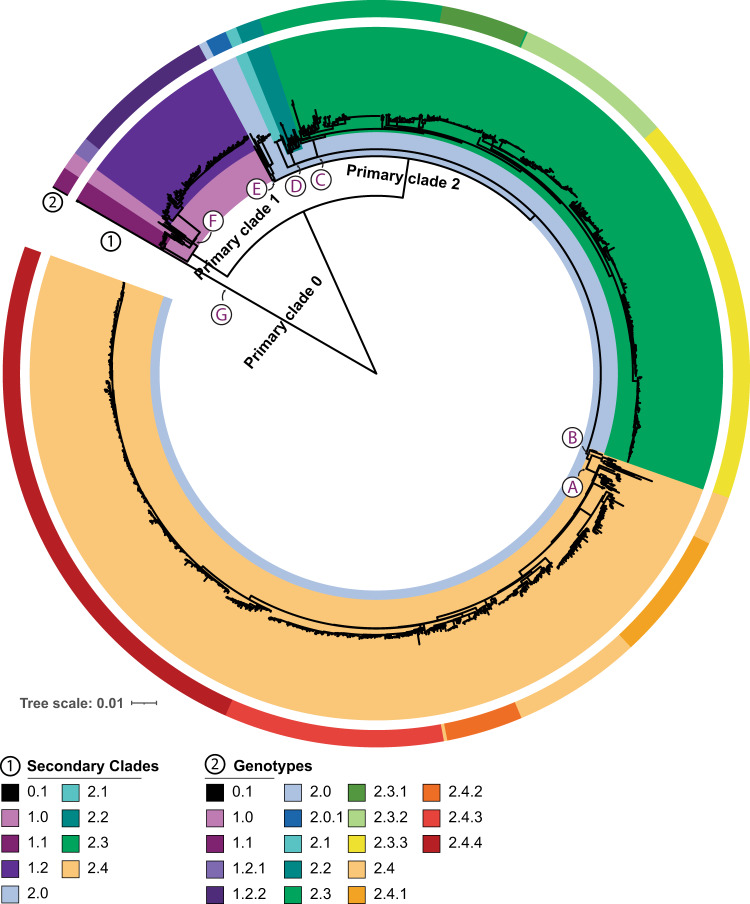
Genotyping scheme for *Salmonella* Paratyphi A. The scheme is composed of three primary, nine secondary and 18 genotypes on a phylogenetic tree of 1379 isolates. The 9 secondary clades as highlighted by the coloring of the inner ring. 18 genotypes identified and are shown in the colored outer ring of the figure. The ancestral nodes for the previously proposed lineage A-G are also shown.

To investigate the genomic diversity of *Salmonella* Paratyphi A, we performed reference-mapping of raw fastq reads and identified 8346 single nucleotide polymorphisms (SNPs) in the 1379 isolates as described earlier^15^. Briefly, all genomes were mapped using bowtie2 to identify the candidate SNPs, then further filtered based on SNP quality (phred >20, homozygous, unambiguous, and unbiased) and location (absent in phage and recombinant regions). This SNP alignment was used in RAxML^16^ to generate a Maximum-likelihood phylogenetic tree of the global collection of *Salmonella* Paratyphi A isolates (Fig. 1). A previously reported lineage scheme, proposed for *Salmonella* Paratyphi A by Zhou et al.^5^ and extended by subsequent studies was overlaid on the phylogenetic tree^6,7,9,17,18^. Visual inspection of the RAxML tree highlighted the insufficiency of the lineage scheme proposed by Zhou et al to fully capture the diversity of *Salmonella* Paratyphi A present. First, while the isolates from lineages B & D - G clustered together, 22 isolates previously assigned to lineages A and C did not. Second, some sequences belonged to clades that diverged from isolates before the exitance of the most recent common ancestor for lineages A and B, indicating that these isolates should be in a different lineage. Third, there was no detailed script of this system with defined SNP or allele for individual lineages. Thus, we tried to assign the lineages based on lineage-majority cluster at the root of secondary or sub-clades of the RAxML tree, and primary lineages A-G could be assigned for 1357 of 1379 (98.4%) genomes. However, when looking for unique definition alleles for these lineages, no unique allele could be identified for 506 (37%) of the 1379 genomes including lineage C (Fig. 1). This was not surprising considering that at the time when Zhou et al. devised this scheme, there were a limited number of sequenced *Salmonella* Paratyphi A genomes available, particularly from South Asia.

**Fig. 2:**
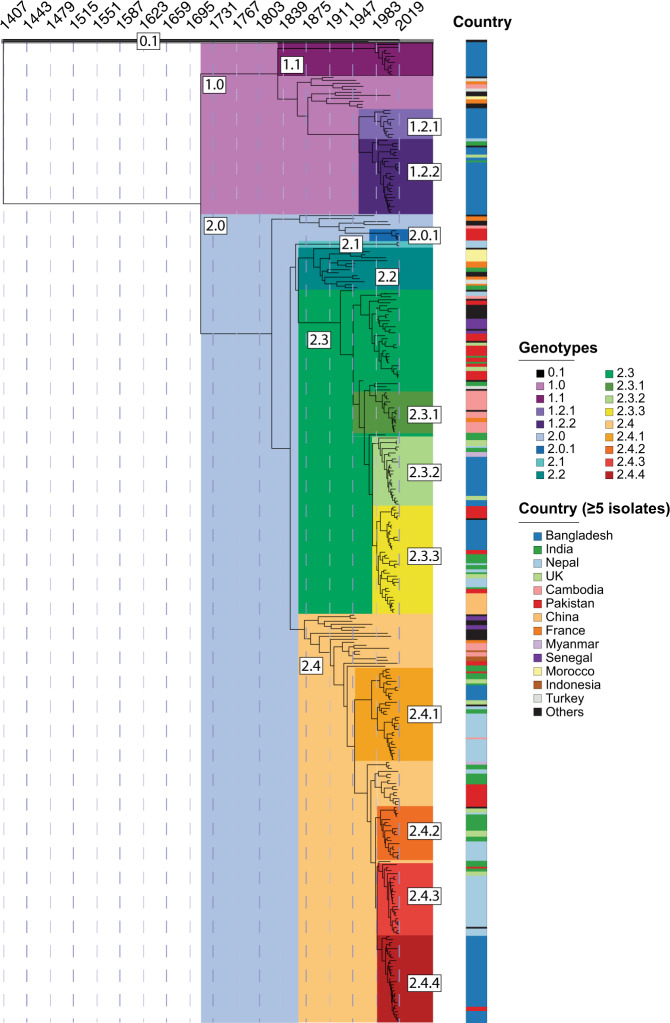
Maximum clade credibility tree of 315 representative *Salmonella* Paratyphi A isolates. The tree shows the last common ancestor of all *Salmonella* Paratyphi A existed at least 600 years ago (tMRCA − 1407 AD). The different genotypes are temporally resolved. Countries with greater than or equal to 5 isolates are also included.

To build a genotyping scheme based on a larger number of representative samples, first, we used fastBAPS^19^ to generate a potential list of clusters in the RAxML tree (Supplementary Fig. 2). Next, we randomly selected a set of 315 isolates from the complete isolate library of 1379, considering two isolates per year for all fastBAPS clusters, and performed phylodynamic analysis using the Bayesian Evolutionary Analysis by Sampling Trees (BEAST) software (Fig. 2, Supplementary Fig. 3). Based on these analyses, we devised a genotyping scheme with three primary clades, nine secondary clades, and 18 genotypes that have circulated globally in the last 100 years.

To aid further genomic epidemiological studies, we identified 18 additional alleles (Supplementary Table 3) that are unique to each of the 18 *Salmonella* Paratyphi A genotypes. These alleles were present in conserved genes involved in essential cellular functions such as protein synthesis, DNA replication, or metabolism and were selected from 2335 genes present in all *Salmonella* Paratyphi A genomes, as identified by the pan-genome analysis. Identification of these genotype-specific alleles allowed us to write a Python script – “*Paratype*” – that assigns genotypes to *Salmonella* Paratyphi A genomes using fastq, bam, vcf, or fasta files obtained during whole genome sequencing (Illumina or nanopore platform) and variant calling The *Paratype* software tool (available at: https://github.com/CHRF-Genomics/Paratype/) has 100% sensitivity and specificity and was able to assign the correct genotype to all the 1379 genomes that were present in our database. Fastq is the slowest but 100% accurate mode of *Paratype*; the fasta mode is slightly less accurate in comparison, 99.78% (1376/1379), but more than 50 times faster (214.05 vs 3.9 s; Supplementary Table 4).

Next, we aimed to extend *Paratype* to include sequences obtained from MinIon platform as long-read sequencing is extensively used in many sequencing laboratories. 33 isolates from the 6 different genotypes were subjected to sequencing on the MinIon R9 flow cell and yielded reads with >20X coverage. The results were run through *Paratype* (*–-mode nano*) and compared with results obtained from Illumina sequencing (*–-mode fastq*) of the corresponding isolates (Supplementary Table 5). Complete genotypes could be assigned to 30/33 genomes, and for these 30 genomes the assigned genotypes were 100% concordant to that obtained from the Illumina platform. For three genomes, while the correct primary (2.0) and secondary (2.4) clade could be determined, the genotype could not be assigned due to an ambiguous base call at the allele positions (Supplementary Table 5).

### Temporal and geographic distribution of different genotypes

**Fig. 3:**
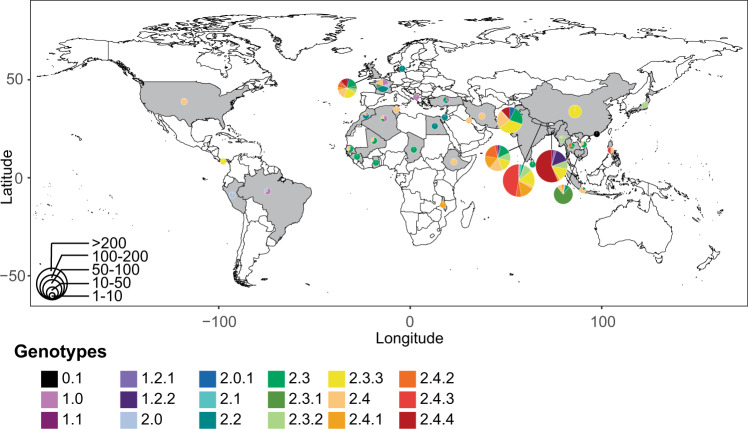
Geographical distribution of *Salmonella* Paratyphi A genotypes. The country of isolation for 1378 sequenced *Salmonella* Paratyphi A isolates is shown. The distribution of genotypes per country is shown as scattered pie charts. The size of each pie chart represents the number of sequences available. A difference in circulating genotypes is observed indicating local populations differ in several endemic countries. Further details are provided in Supplementary Data 1.

Upon the establishment of the “*Paratype*” scheme, we considered the geographical distribution of the different genotypes (Fig. 3). Genotype 0.1 under primary clade 0 was phylogenetically unique (matches with lineage H of Zhou et al.^5^); there was only one isolate belonging to this genotype/primary clade that was isolated in Hong Kong in 1971. The genome of this isolate was distinct from all other genomes obtained thus far, contained 1288 unique SNPs, and may represent a lineage that is now extinct, or present at very low numbers in areas that have not been sampled. The other two primary clades, clades 1 and 2 contain genomes that have been collected in the last two decades and from the Bayesian analysis, these two clades appear to have emerged between 1700 and 1800. However, as most sequences were obtained from recently collected isolates, the error in this estimate is likely to be high. Clade 1 contains genomes largely from lineage F of the previous lineage scheme, and fastBAPS predicted two sub-clusters within this clade. One of these clusters was largely found in Bangladesh and has been assigned secondary clade 1.2, then sub-divided into genotypes 1.2.1 and 1.2.2 which appear to have diverged in the 1950s. Both these genotypes are currently present in Bangladesh and other South Asian countries (Fig. 2). The other cluster with 13 genomes from Bangladesh that were first isolated in 1999 have been assigned to genotype 1.1. The remaining 10 genomes were obtained between 1917 to 1963 and have been assigned genotype 1.0.

Most *Salmonella* Paratyphi A genomes (1254/1379; 91%) have been assigned to primary clade 2, which contains genomes belonging to the lineage A-E of the previous scheme. Genomes that belonged to lineages B, D, and E have now been assigned to genotypes 2.4, 2.2, and 2.0, respectively. Within genotype 2.0, 13 unique and recent isolates from Pakistan were identified and have been assigned as genotype 2.0.1. Genotype 2.1 contains isolates from Nepal that were sampled during the SEAP study, yet the genotype emerged in the 1800s and is distinct from all other isolates in clade 2. Two clusters in fastBAPS, comprising of strains largely from what was formerly C lineage are now assigned to genotype 2.3. Genotype 2.3 has been subdivided into genotypes 2.3.1 to 2.3.3, each of which belongs to a distinct geographical location: 2.3.1 is found predominantly in South-East Asia; 2.3.2 and 2.3.3 are found largely in South Asia. An outbreak of paratyphoid fever in China during 2010–2011^7^ was caused by isolates of genotype 2.3.3, and these likely originated in South Asia. The former lineages A and B have been assigned genotype 2.4, which is further divided into 2.4.1 to 2.4.4. While genotypes 2.4.1 and 2.4.2 have been observed in different countries in South Asia, genotype 2.4.4 is predominantly found in Bangladesh, and 2.4.3 is largely present in Nepal.

Different countries in South Asia had unique genotype distributions. Predominant genotypes present in Bangladesh were 2.4.4 (56%) followed by 1.2.2 (14%) and 2.3.3 (13%). In Nepal, 2.4.3 (47%), 2.3.3 (16%) and 2.4.1 (14%) were three most common genotypes. Pakistan had genotypes 2.3.3 (25%), 2.3 (16%) and 2.4 (15%). In India, genotypes 2.4.2 (22%), 2.4 (20%), 2.4.1 (19%), 2.3.3 (17%), and 2.3 (16%) were commonly identified.

### Antimicrobial resistance markers in *Salmonella* Paratyphi A

**Fig. 4:**
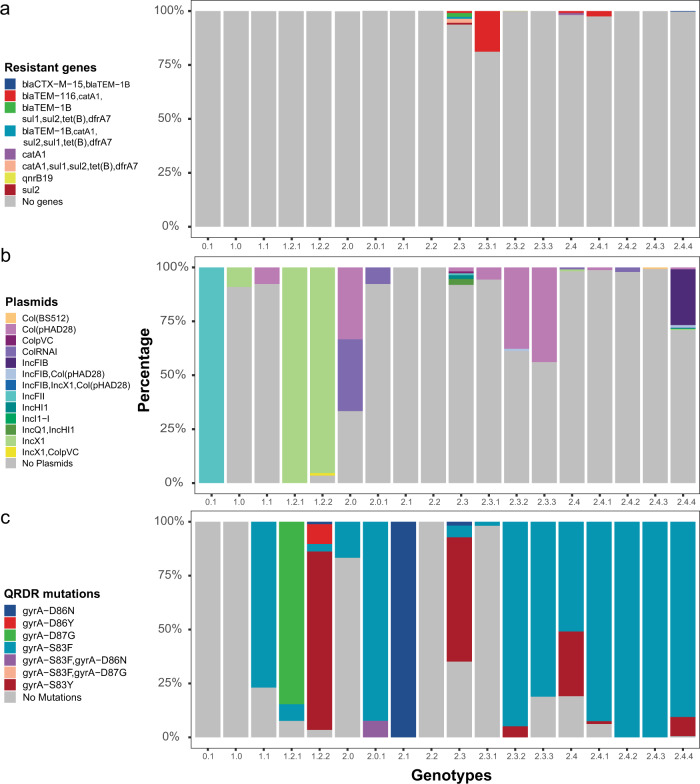
Presence of antimicrobial resistance genes, plasmids, and chromosomal mutations linked to quinolone resistance across different *Salmonella* Paratyphi A genotypes. The diversity of (**a**) Antimicrobial resistance genes (**b**) Plasmids and (**c**) Quinolone resistance determining region (QRDR) mutations present *Salmonella* Paratyphi A is shown.

To characterize genomic determinants of antimicrobial resistance in *Salmonella* Paratyphi A, we screened the 1379 genomes for the presence of antimicrobial genes and markers using ResFinder^20^ (Fig. 4a) and plasmids using PlasmidFinder^21^ (Fig. 4b). Of the 1379 isolates, 1356 (98%) had no predicted antimicrobial resistance genes and 1015 (74%) isolates showed no predicted plasmids. Five genomes with the IncHI1 plasmid were identified; two genomes (both from India) contained resistance genes for trimethoprim and chloramphenicol, and the other three genomes contained genes for trimethoprim, chloramphenicol, and ampicillin designated as MDR isolates (one each from India, Pakistan, and Thailand). All five genomes belonged to genotype 2.3 and the strains were isolated between 1999–2004. We also identified a genome belonging to genotype 2.4.4 containing *bla*_CTX-M-15_ and *bla*_TEM-1B_ on an IncI1-I plasmid; the originating strain was isolated from a patient who contracted the infection in Bangladesh in 2017^22^. There were 14 isolates including 10 from the genotype 2.3.1 that contain *bla*_TEM-116_, which can lead to resistance to ampicillin; all 14 were reported from Cambodia^6^. Another isolate from genotype 2.3.3 (from Pakistan, 2015) contained a *qnrB19* gene on a Col(pHAD28) plasmid, which has been shown to lead to quinolone resistance in other *Salmonella* species^23^.

In addition to antimicrobial resistance genes, we searched for chromosomal mutations in the *acrB* gene and the quinolone resistance determining region (QRDR) to identify isolates resistant to azithromycin and ciprofloxacin, respectively. Six of 1379 genomes contained an AcrB-R717 mutation, all from Bangladesh and these belonged to genotypes 2.3.3 (1/6) and 2.4.4 (5/6)^15,24^. The first azithromycin-resistant *Salmonella* Paratyphi A isolate was identified in 2014, and this resistance has emerged independently at least twice in two different genotypes. On the other hand, a majority (1174/1379; 85%) of genomes had mutations in the QRDR region. The most common single mutation was gyrA-S83F (938/1379), followed by gyrA-S83Y (205/1379). Two isolates contained double mutations in the QRDR region; one of them belonged to genotype 2.0.1 (gyrA-S83F & D87N, Pakistan, 2017) and another belonged to genotype 2.3.3 (gyrA-S83F & D87G, UK, 2016). Barring genotype 0.1, 1.0 and 2.2, all other genotypes had at least one genome with a QRDR mutation (Fig. 4c). The first QRDR mutation was identified in 1997 in India in genotype 2.4 and their prevalence has increased over time. In 2012 and 2013, there was an outbreak in Cambodia caused by a strain from genotype 2.3.1 that did not have any QRDR mutation leading to a temporary increase in proportion of *Salmonella* Paratyphi A with no QRDR mutations during these two years (Supplementary Fig. 4).

To validate the genomic observation, we abstracted results of antimicrobial susceptibility testing for the 528 isolates from Bangladesh against ampicillin, chloramphenicol, cotrimoxazole, ceftriaxone, ciprofloxacin and azithromycin from available electronic records at the Child Health Research Foundation. Concordant with the results obtained from *Paratype*, no isolate was resistant to ampicillin, chloramphenicol, cotrimoxazole or ceftriaxone. Five isolates exhibited azithromycin resistance in complete concordance with *Paratype* results, which detected the AcrB-R717 R > Q mutation responsible only in these isolates. 523 of 528 isolates were phenotypically non-susceptible to ciprofloxacin, of which *Paratype* detected at least one QRDR mutation in 520 isolates. All results are available in Supplementary Data 1.

## Discussion

*Salmonella* Paratyphi A is the causative agent of paratyphoid fever, a neglected tropical disease with a high burden in low-and-middle-income countries. Limited information is available regarding its genomic diversity, especially from South Asian countries that collectively are responsible for over 80% of all paratyphoid cases. As genomic surveillance becomes more prominent, there is a need for a coherent and easy-to-use scheme that can be deployed by public health researchers and do not require extensive computing resources or expertise.

We sequenced a total of 817 isolates originating from Bangladesh, Nepal and Pakistan collected over the last 20 years and compiled a collection of all genomes of *Salmonella* Paratyphi A publicly available thus far. We describe a genotyping framework for *Salmonella* Paratyphi A using 1379 isolates obtained from 1917 through 2019. Rather than being guided by a single approach, we combined maximum likelihood-based phylogenetics with BAPS and Bayesian analysis via BEAST to design a genotyping scheme for *Salmonella* Paratyphi A. The scheme divided the *Salmonella* Paratyphi A population into 18 different genotypes, and each can be identified by the presence of an allele that is located on the coding sequence of a conserved gene, involved in housekeeping functions. We only found 8346 SNPs from all 1379 isolates, with minimal recombination, and thus, this genotyping scheme based on SNP alleles can support robust genotyping and accommodate future evolution of *Salmonella* Paratyphi A. And to assist with that, we have developed *Paratype*, an open-source Python script for genotyping of *Salmonella* Paratyphi A genomes. *Paratype* can detect the genotype of *Salmonella* Paratyphi A genomes directly from raw fastq reads of both Illumina and Nanopore platforms or processed fasta, bam, or vcf files. It can also detect mutations in the *acrB* efflux pump (determinant of macrolide resistance) and the QRDR region (determinant of ciprofloxacin non-susceptibility).

In this genotyping scheme, we propose three primary clades 0, 1, and 2, which diverged before the 1800s (Fig. 2). While only a single isolate of primary clade 0 was obtained in 1971, isolates belonging to clade 1 and 2 have been routinely identified over the past two decades. Clade 2 is the most abundant and has been subdivided into four secondary clades: 2.1–2.4, which emerged in the 1800s. Clade 2.3 could be subdivided into 2.3.1–2.3.3, each with distinct geographic distribution. Clade 2.4 was also sub-divided into genotypes 2.4.1–2.4.4. Genotype 2.4.4 was the most abundant and was predominantly present in Bangladesh. This genotype emerged in the early 1990s and possesses high rates of ciprofloxacin non-susceptibility (Figs. 2 and 4). Five of the isolates from this genotype also contained AcrB-R717Q mutation that leads to azithromycin resistance, while one was found to harbor a plasmid containing extended-spectrum beta-lactamase gene (*bla*_CTX-M-15_)^22^.

In line with findings of previous studies, the rates of acquisition of antimicrobial resistance markers in *Salmonella* Paratyphi A are lower relative to *Salmonella* Typhi (Fig. 4)^6,9^. Although a few isolates acquired the IncHI1 plasmid in the late 1990s to early 2000s (Fig. 4a), no massive spread across the globe was noted; this is unlike *Salmonella* Typhi lineage H58 (genotype 4.3.1) carrying a similar IncHI1 plasmid that spread and became the dominant lineage in the last 30 years^25^. This is also true for chromosomal mutations such as QRDR and AcrB mutations, which are overall less prevalent in *Salmonella* Paratyphi A than in *Salmonella* Typhi^25,26^. Considering the genetic similarities between *Salmonella* Typhi and Paratyphi A, and the fact that they occupy the same environmental niche, the differences in the presence of AMR genes between these typhoidal *Salmonella* serovars warrants further investigation.

As more genomes are added to the database, we will continue updating *Paratype*. One feature of public health interest would be the genomic prediction of the O2-antigen in *Salmonella* Paratyphi A. Most of the vaccines being developed for *Salmonella* Paratyphi A use the O2-antigen that is unique to this serovar conjugated to a carrier protein^27^. Recently, through in-silico metabolic reconstruction, an 18.9 kb region containing genes involved in O-antigen biosynthesis was identified as important for determining the specific molecular features of the O2-antigen found in *Salmonella* Paratyphi A^28^. At present there is little data linking genetic variation with the O2-antigen chemistry. However, as the vaccine development progresses, all mutations in this region will need to be carefully monitored and *Paratype* can assist in that effort.

*Paratype* will be updated at least twice a year by screening NCBI, ENA, and Enterobase to identify new *Salmonella* Paratyphi A genomes. In addition, we will also keep track of user notifications about new genomes that do not fall in the predicted genotypes of the latest version. If more than 10 genomes are obtained where genotypes are unassigned, we will generate a phylogenetic RAxML tree including the additional genomes and check for the presence of new clusters. If a new cluster is indeed identified, using the methodology described here, we will identify the unique alleles for the cluster, and assign a new genotype. For example, if 10 new genomes that were previously assigned to 2.3 clusters together in the new phylogenetic tree, we can assign them to 2.3.N.

The conclusions that we can draw from this analysis are subject to certain limitations. First, *Salmonella* Paratyphi A is a neglected pathogen, and hence the available genomes, might lack broad representativeness across geographies or time. Specifically, a small proportion of genomes were available from countries in sub-Saharan Africa and India. Additionally, most sequences were from isolates collected within the last two decades. Second, while the tool has high sensitivity and specificity to our dataset, as more genomes become available over time and novel mechanisms of AMR emerge, this tool will require updates from the bigger scientific community. Like all genotyping tools, *Paratype* is a living tool that will require updates. Our diverse group of authors plans to continually monitor the library of publicly available genomes, accept update requests via GitHub, and incorporate any required updates in the *Paratype* scheme accordingly.

In summary, in this study we present a large-scale analysis of *Salmonella* Paratyphi A genomes and propose a genotyping tool for this pathogen. We released *Paratype* (https://github.com/CHRF-Genomics/Paratype) as an open-access tool that can use sequences from both Illumina and Nanopore platforms. It is an easy-to-use, command-line tool, which is being tested and adopted by researchers for genomic analysis. This tool will assist future genomic surveillance studies and will help inform prevention and treatment strategies for this neglected pathogen.

## Methods

### Inclusion and ethics

Ethical approval for the parent studies at CHRF (that includes the sequenced isolates) was obtained from the Bangladesh Institute of Child Health Ethical Review Committee. In addition, for the SEAP isolates from Nepal and Pakistan, ethical approvals were taken from Nepal Health Research Council, and Aga Khan University Hospital Ethics Committee and Pakistan National Ethics Committee. For the hospitalized cases, informed written consent and clinical information were taken from adult participants and legal guardians of child participants. No compensation was provided to the participants.

### Study site and isolate selection

This study includes genomic data generated from Bangladesh, Nepal and Pakistan.

Bangladesh: The Child Health Research Foundation in Bangladesh has been preserving invasive *Salmonella* isolates since 1999 and maintains a biobank of >9000 typhoidal *Salmonella* isolates, largely from children (<18 years of age) that were isolated from the blood of the patients in two different settings: in-patient (hospitalized), and out-patient (community) facility^29^. Clinical and epidemiological data were collected for all hospitalized patients. From a biobank of 1123 *Salmonella* Paratyphi A isolates collected till June 2018, 528 were randomly selected for whole-genome sequencing (WGS) considering the year of isolation, gender, collection sites, and hospitalization settings (Supplementary Table 1). Of these, 180 isolates were collected and sequenced under the Surveillance for Enteric Fever in Asia (SEAP) project in Bangladesh during 2016–2018.

Nepal and Pakistan: SEAP was also conducted in two other typhoid-endemic countries, Nepal and Pakistan and 156 isolates from Nepal and 133 isolates from Pakistan were sequenced and added to this study. The SEAP-Nepal isolates with WGS data included all pre-SEAP isolates (2014–2016) and randomly selected SEAP isolates (2017–2019). The SEAP-Pakistan isolates with WGS (*n* = 133) were selected prioritizing the availability of geographic information and susceptibility profile during 2016–2018.

In total, 817 *Salmonella* Paratyphi A genomes were generated from these three typhoid-endemic countries.

To add to all the isolates sequenced in this study, we also collected raw fastq data of 560 *Salmonella* Paratyphi A isolates from 37 different countries and 10 published articles (Supplementary Table 2). Complete chromosomal sequences of *Salmonella* Paratyphi A ATCC 9150 (https://www.ncbi.nlm.nih.gov/assembly/GCF_000011885.1) and AKU_12601 (https://www.ncbi.nlm.nih.gov/assembly/GCA_006518435.1) were also included^30,31^. For travel-related paratyphoid cases, the country of “traveling from” was considered as the country of origin. If no travel data were available, the country of “reported from” was considered as the country. Overall, for globally distributed 562 *Salmonella* Paratyphi A, year and country data were available for 507 and 536 respectively (Supplementary Table 2). In total, we obtained a global collection of 1379 *Salmonella* Paratyphi A covering a timeline of 1917–2019 and 37 countries (Supplementary Data 1 for more details).

### Antimicrobial susceptibility testing

Results of antimicrobial susceptibility testing using the Kirby Bauer disk diffusion method for ampicillin, chloramphenicol, cotrimoxazole, azithromycin and ciprofloxacin were abstracted from available electronic records at the Child Health Research Foundation for the 528 genomes from Bangladesh. Isolates that were resistant to azithromycin by the disc diffusion method, were retested using MIC strips (bioMérieux, Marcy-l’Étoile, France). Zone diameter and MIC results were interpreted according to the latest Clinical Laboratory Standard Institute (CLSI) guidelines.

### Whole-genome sequencing

*Salmonella* Paratyphi A isolates from 1999–2016 from Bangladesh (*n* = 348) were sub-cultured on MacConkey agar media and kept overnight at 37 °C. In case of any visible contamination, a single colony was picked and subcultured again. Later, all colonies were swabbed and resuspended into 1 ml of molecular grade water. From this suspension, 400 µL was used for DNA extraction using the QIAamp DNA Mini Kit (Qiagen, Hilden, Germany) and sent to Novogene (NovogeneAIT, Singapore) for WGS on Novaseq 6000 platform (PE150). All SEAP isolates (including the 180 from Bangladesh) were extracted using the same protocol and were sequenced on Illumina HiSeq X Ten platform (PE150) at the Wellcome Sanger Institute, Cambridge, UK.

For long-read sequencing, we used the Oxford nanopore technology (ONT) sequencing platform. The nanopore libraries were generated using the genomic DNA of 33 randomly-selected isolates (from 8 different genotypes) by following the Rapid sequencing DNA-PCR barcoding protocol (SQK-RPB004). Whole genome sequencing was performed using a MinION MK1B device (R9 flow cell) using the MinKNOW platform (72 h) at the CHRF laboratory in Bangladesh. Fast5 data files were base-called using the guppy basecaller v6.0.1 and demultiplexed using qcat v1.1.0 (https://github.com/nanoporetech/qcat).

### Systematic literature review of existing *Salmonella* Paratyphi A genomes

To contextualize the genomes sequenced in this study, we conducted a systematic search to compile all publicly available *Salmonella* Paratyphi A genomes (for which raw reads and metadata were available) to build a database of 560 additional isolates from 10 studies (Supplementary Table 2). First, the search terms “(Salmonella Paratyphi A) AND (Molecular Epidemiology)” “Salmonella Paratyphi A genome” and “(Salmonella Paratyphi A) AND (Genomic Epidemiology)” were used in PubMed advanced search builder. Next, the hits were filtered by selecting dates between 1900 and 2019 and the total number of publications remaining was 231. After screening the abstracts and titles manually and eliminating duplicates, only 7 studies were found to have any kind of genome/metadata available for further analysis. In addition, three studies^8,9,22^ that meet our criteria (published and both metadata and raw reads available) but missed/not published during the initial PubMed search were incorporated from European Nucleotide Archive (ENA) database, taking the final number of incorporated publications to 10.

### Quality check, genome assembly, annotation, and pan-genome analysis

Raw Illumina fastq reads of all *Salmonella* Paratyphi A were quality-checked using FastQC v0.11.5 and trimmed using Trimmomatic if necessary^32^. All sets of raw sequencing reads obtained from Illumina and ONT were assembled using Unicycler v0.4.8 (*default with –min_fasta_length 200*)^33^. The assembled contigs (*n* = 1377) and downloaded complete chromosomes (*n* = 2) were annotated using Prokka (*--gcode 11 --mincontiglen 200*)^34^. The annotated GFF files of all 1379 isolates were used to build a pan- and core-genome of *Salmonella* Paratyphi A using Roary v3.3 (*options: -t 11 -e --mafft -n*)^35^. The gene_presence_absence matrix output was used to perform the Heap’s law analysis to understand the open/closedness of the pan-genome (*heaps* function of *micropan* library on R; 1000 permutations).

### SNP-based phylogenetic analyses

For the complete “global+SEAP” raw data collection, Illumina fastq reads of 1377 *Salmonella* Paratyphi A and fasta of two RefSeq chromosomes (NC_006511 (https://www.ncbi.nlm.nih.gov/assembly/GCF_000011885.1) and NC_011147 (https://www.ncbi.nlm.nih.gov/assembly/GCA_006518435.1)) were mapped against the reference *Salmonella* Paratyphi A AKU_12601 (https://www.ncbi.nlm.nih.gov/nuccore/NC_011147.1) using Bowtie2 v2.3.5.1^36^. The reference genome, AKU_12601 (https://www.ncbi.nlm.nih.gov/assembly/GCA_006518435.1) is a part of the NCBI Refseq database and was isolated from a paratyphoid patient in Karachi, Pakistan in 2002. Candidate SNPs were identified using SAMtools (v1.10) and BCFtools (v1.10.2)^37^. Only the homozygous, unambiguous SNPs with a Phred-quality score of >20 were selected using a customized Python script. SNPs were discarded if they had strand bias *p* < 0.001, mapping bias *p* < 0.001 or tail bias *p* < 0.001 (using vcfutils.pl script, from SAMtools). SNPs located in phage or repeat regions (118.9 kb for *Salmonella* Paratyphi A AKU_12601 as described in Sajib et al.^15^) were also excluded using a customized python script. Gubbins v2.3.4 was used to detect the recombinant regions^38^ and SNPs in those regions were excluded as well using the same python script, resulting in a set of 8346 chromosomal SNP positions for the “global+SEAP” collection (*n* = 1379). All SNP alleles were extracted (fasta) using a customized python script and merged to produce SNP alignment.

Maximum likelihood trees (MLT) were built from the chromosomal SNP alignments using RAxML v8.2.12 (with the Generalized Time-Reversible model and a Gamma distribution to model site-specific rate variation; GTRGAMMA in RAxML)^16^. Support for the MLT was calculated using 100 bootstrap pseudo-analyses of the alignment. The MLT was outgroup rooted by including the pseudo-alleles from *Salmonella* Typhi CT18 (https://www.ncbi.nlm.nih.gov/nuccore/NC_003198.1) in the alignment. Tree visualization was done using iTol v5.5^39^, including the previous Paratyphi A lineages proposed by Zhou et al.^5^.

### Bayesian analysis and identifying phylogenetically informative clades and subclades

In addition to SNP-based MLT, we investigated the population structure of the global *Salmonella* Paratyphi A collection using a Bayesian approach, implemented with the SNP alignment using fastBaps^39^. To maintain compatibility with the phylogeny, some minor modifications were made to the clustering pattern proposed by the least conservative Dirichlet prior hyperparameters on fastbaps, *optimise.baps*. This eventually resulted in a total of 16 different clusters. A customized python script was used to randomly select two isolates/year/cluster to represent this global collection of *Salmonella* Paratyphi A, leading to two independent sample sets of 315 isolates each. The alignment of SNP-alleles for this representative sample set was used to understand the evolutionary diverging pattern of different *Salmonella* Paratyphi A clusters over time using BEAST v1.10.4^40^. The GTR + Γ(4) substitution model was selected for this analysis with the exponential unrelated relaxed clock as clock type and Bayesian skyline coalescent model as tree prior. The analysis considered the year of isolation as tip dates and continued for 500 million steps with sampling every 50,000 iterations. The BEAST analysis was run twice each on the two independently generated sets of isolates. The resulting log files and model parameters were analyzed on Tracer v1.7.1. TreeAnnotator v1.10 was used to generate the maximum–clade-credibility (MCC) tree^41^. The tree was visualized on FigTree v1.4.4 with a time scale. For the model with the highest posterior values (joint effective sample size (ESS) of 544) used for further analysis, time to last common ancestor (tMRCA) was calculated to be 1407 AD (95% highest posterior density (HPD) interval [721.0, 1637.3]). Based on the diverging patterns suggested by the MCC tree, we assigned the clusters (defined as described above) into primary clades, secondary clades, and subclades on the MLT. However, a few visible clusters on the MLT could not be assigned to specific subclades due to a lack of clustering information from fastBaps, likely due to the low number of SNPs unique to these clusters.

### SNP-based genotyping scheme and *Paratype*

We further divided the 16 clusters obtained from fastBAPS into 18 genotypes and identified a set of 18 SNP alleles, located in a coding sequence for conserved genes to define each assigned secondary clade and subclades. Each SNP allele was unique to only one subclade or, to one secondary clade and its corresponding subclades (if any). Therefore, we assigned the term “genotype” to each of the 18 secondary clades or subclades. Sorted read alignment (BAM) files generated during the SNP analysis were used to assign the genotypes for each isolate using a customized Python script, named *Paratype* (available at https://github.com/CHRF-Genomics/Paratype). Briefly, under the default BAM mode *(--mode bam*), *Paratype* uses *samtools index* (if bam file is not indexed), *samtools mpileup*, and *bcftools call* to extract the consensus base calls at those 18 SNP loci from the BAM file. The resulting variant call format (VCF) file is then processed to identify the presence of the defining SNP alleles and follow cladistic logic to assign the genotype of the isolate, as well as the primary clade, secondary clade, and subclade information. *Paratype* only considers high-quality SNP alleles (Phred score >20 and 75% read_ratio for the allele) to assign genotypes. Read_ratio is calculated by the number of high-quality alternative-allele reads on both strands, divided by the total number of high-quality reads. In addition, *Paratype* also has fastq, (*--mode fastq*), fastq interleaved (*--mode fqin*) and nano (*--mode nano*) modes, where a user can provide a set of paired-end or interleaved Illumina or Nanopore fastq data file (can be gzipped) and *Paratype* performs reference mapping (against the *Salmonella* Paratyphi AKU_12601 (https://www.ncbi.nlm.nih.gov/nuccore/NC_011147.1) genome) using Bowtie (or, BWA) and SAMtools and follows the same steps described above to detect the genotype of the isolates. Although the bam mode is the default for the tool, the *fastq*, *fqin* and *nano* modes are more accurate and should be user-friendly to non-coding specializing researchers; however, it is more time-consuming. *Paratype* also runs on fasta (*--mode fasta*) and vcf mode (*--mode vcf*). Both of them are faster, but vcf mode is also the least accurate if the provided SNPs are not highly trusted.

### Plasmid, resistance gene, and mutation analysis

All assembled contigs were screened with PlasmidFinder v2.1^21^ and ResFinder v3.2^20^ to detect plasmid amplicons and acquired AMR genes respectively. Both results were parsed using customized python scripts. To detect mutations in *gyrA* and *acrB* genes, we used the same *Paratype* script. It uses the same files used for genotyping and produces gene- and position-specific non-silent and silent mutation results.

### Data visualization and statistical analysis

R (v4.0.4) base function and several packages including dplyr, ggplot2, micropan and scatterpie were used for data visualization and statistical analysis.

### Reporting summary

Further information on research design is available in the Nature Portfolio Reporting Summary linked to this article.

## Supplementary information

Supplementary Information

Description of Additional Supplementary Files

Supplementary Dataset 1

Reporting Summary

## Data Availability

The raw reads (both Illumina and ONT) of 528 *Salmonella* Paratyphi A isolates from CHRF, Bangladesh supporting the conclusions of this article are available in the European Nucleotide Archive (ENA) under study accession ERP132884 (https://www.ebi.ac.uk/ena/browser/view/PRJEB48506) (*n* = 348) and ERP112783 (https://www.ebi.ac.uk/ena/browser/view/PRJEB30334) (*n* = 180; from SEAP). The assembled contigs for all 528 genomes are also available under the study accession ERP132884 (https://www.ebi.ac.uk/ena/browser/view/PRJEB48506). Raw reads of the isolates from the SEAP project in Nepal and Pakistan are also available on ENA under study accession ERP112783 (https://www.ebi.ac.uk/ena/browser/view/PRJEB30334). All accessions are included in Supplementary Data 1. The metadata and antimicrobial susceptibility data supporting the conclusions of this article are also included in Supplementary Data 1. Source data for the figures (including supplementary figures) are provided with this paper. All correspondence and material requests should be addressed to Dr. Senjuti Saha (senjutisaha@chrfbd.org). Source data are provided with this paper.

## Source data

Source Data

## Code availability

The genotyping tool for *Salmonella* Paratyphi A, *Paratype* is available at https://github.com/CHRF-Genomics/Paratype (10.5821/zenodo.7294627). Customized Python scripts and color schemes used in the manuscript are available at https://github.com/CHRF-Genomics/CHRF_Paratyphi_scripts.
