# Supplementary material for: Paratype: a genotyping tool for Salmonella Paratyphi A reveals its global genomic diversity
Source: Nat Commun. 2022 Dec 23;13:7912. doi: 10.1038/s41467-022-35587-6 (PMC9782287; doi:10.1038/s41467-022-35587-6)
Supplement: Supplementary file 1 — Supplementary Information [file 41467_2022_35587_MOESM1_ESM.pdf]

Supplementary Information

Paratype: A genotyping tool for *Salmonella* Paratyphi A reveals its global genomic diversity

Table of content:

Supplementary Figures 2

Supplementary Figure 1: Pan-genome of 1379 *Salmonella* Paratyphi A genomes. 2

Supplementary Figure 2: FastBAPS output for 1379 isolates used for designing the genotyping system. 3

Supplementary Figure 3: Analysis of the phylogenomic tree obtained from BEAST. 4

Supplementary Figure 4: QRDR mutations among global *Salmonella* Paratyphi A isolated between 1917 and 2019. 5

Supplementary Tables 6

Supplementary Table 1: Selection of isolates from the CHRF *Salmonella* Paratyphi A BioBank for whole-genome sequencing. 6

Supplementary Table 2: Summary of 1,379 isolates used in the study. 7

Supplementary Table 3: List of alleles for the 18 genotypes. 8

Supplementary Table 4: Comparison of run time required by different run modes of the Paratype tool. 9

Supplementary Table 5: Comparison of *Paratype* results using raw fastq data from Illumina and Nanopore platforms. 10

Supplementary references 12

Supplementary Figures

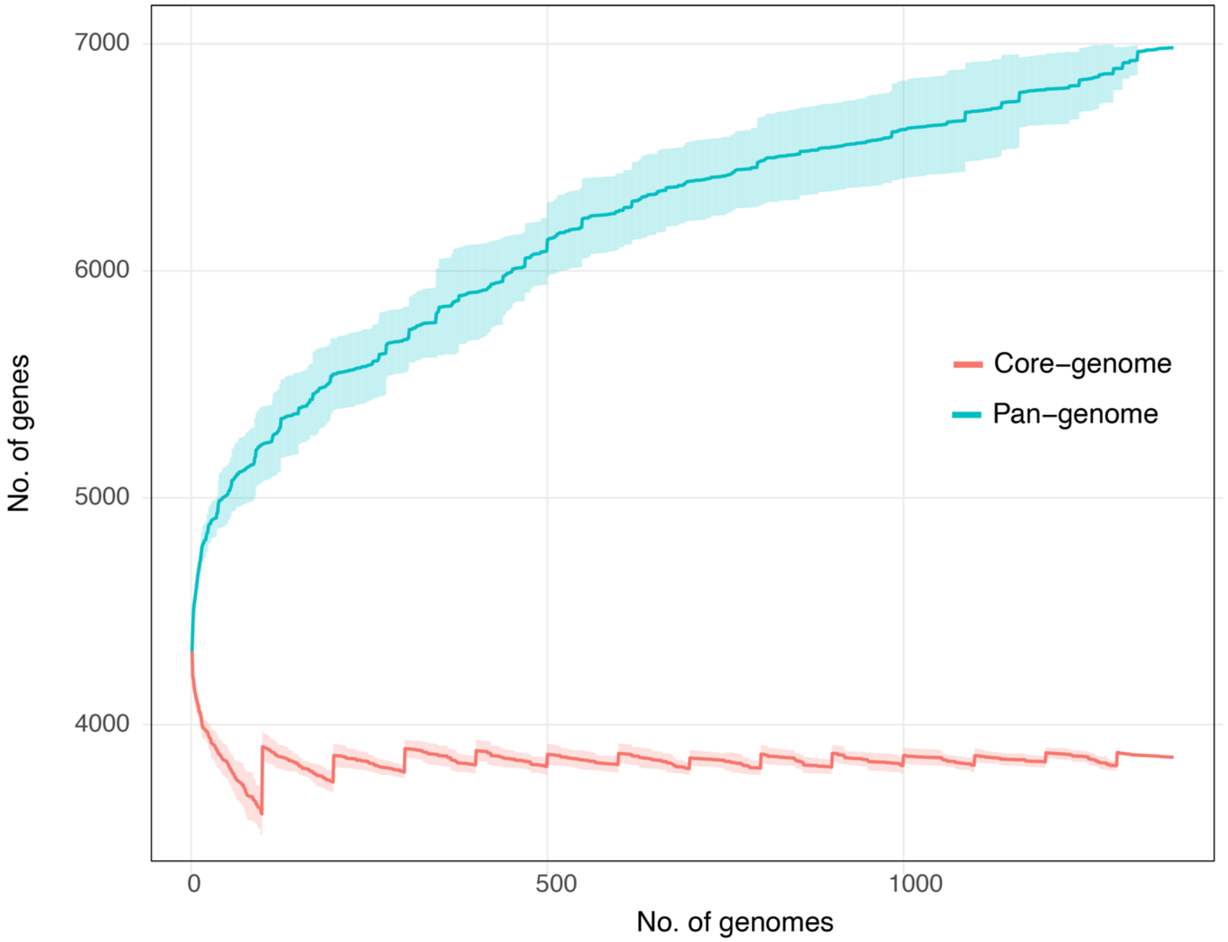

Supplementary Figure 1: Pan-genome of 1379 *Salmonella* Paratyphi A genomes. 6983 unique genes were identified, of which 3857 were genes present in >99% of isolates, 257 genes present in 95-99% of isolates, 319 genes present in 15-95% of isolates), and 2550 genes that were found to be present in less than 15% of isolates. The line shows mean values +/- SD.

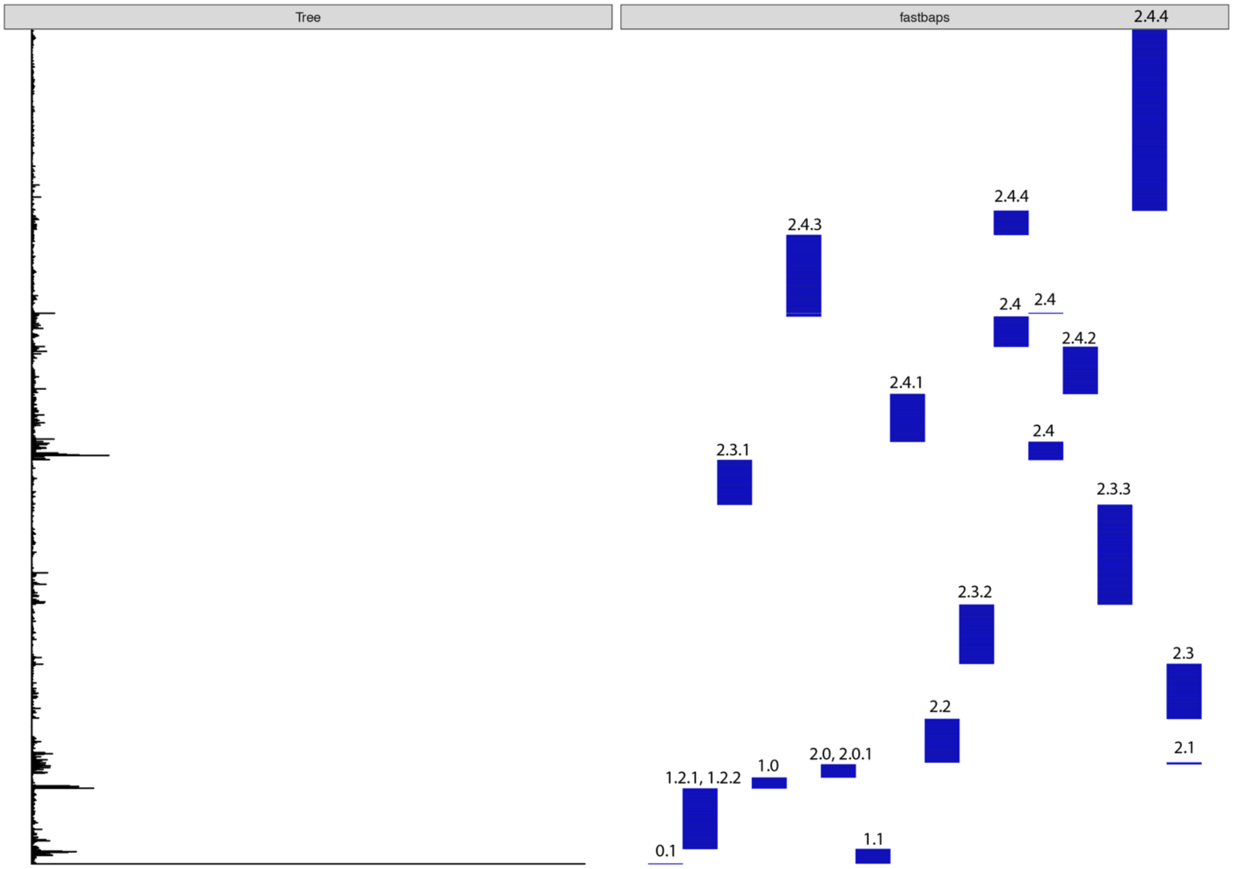

Supplementary Figure 2: FastBAPS output for 1379 isolates used for designing the genotyping system. 16 clusters are obtained using "-optimise.baps" clustering option in fastBAPS. The plot shows the phylogenetic tree obtained from RAxML (tree) with the 16 different clusters obtained (fastbaps). The final assigned genotypes assigned to the different fastBAPS cluster are also listed.

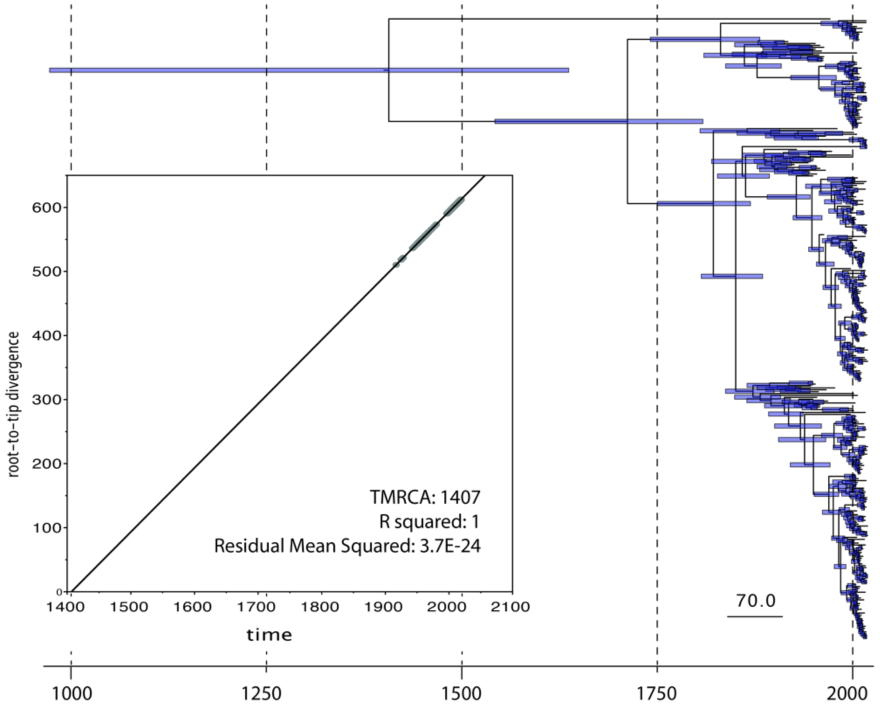

Genotype	Emergence Date (95%HPD interval)
0.1	1407 (973,1637)
1.0	1712 (1542,1809)
1.1	1831 (1741,1881)
1.2.1	1957(1921,1979)
1.2.2	1957(1921,1979)
2.0	1822(1750,1869)
2.0.1	2007(1997,2013)
2.1	1859(1819,1890)
2.2	1863(1827,1894)
2.3	1928(1891,1946)
2.3.1	1973(1961,1985)
2.3.2	1987(1979,1995)
2.3.3	1984(1977,1990)
2.4	1872(1837,1899)
2.4.1	1976(1960,1987)
2.4.2	1990(1983,1996)
2.4.3	1987(1979,1994)
2.4.4	1992(1985,1998)

Supplementary Figure 3: Analysis of the phylogenomic tree obtained from BEAST. The 95% HPD interval for each of the predicted internal nodes is shown with a blue bar. The predicted emergence date and 95% HPD for each of the 18 genotypes in Paratype are listed in the table on the right panel. The maximum clade credibility tree was further analyzed using TempEst v1.5.3. The sampling dates of the genomes were included to estimate the root-to-tip divergence for the all the genomes used in the analysis (Rsquared =1, Residual Mean Squared: 3.7E-24). The time to the most recent common ancestor (TMRCA), predicted by intercept of the fitted model on the X-axis, was estimated to be 1407.

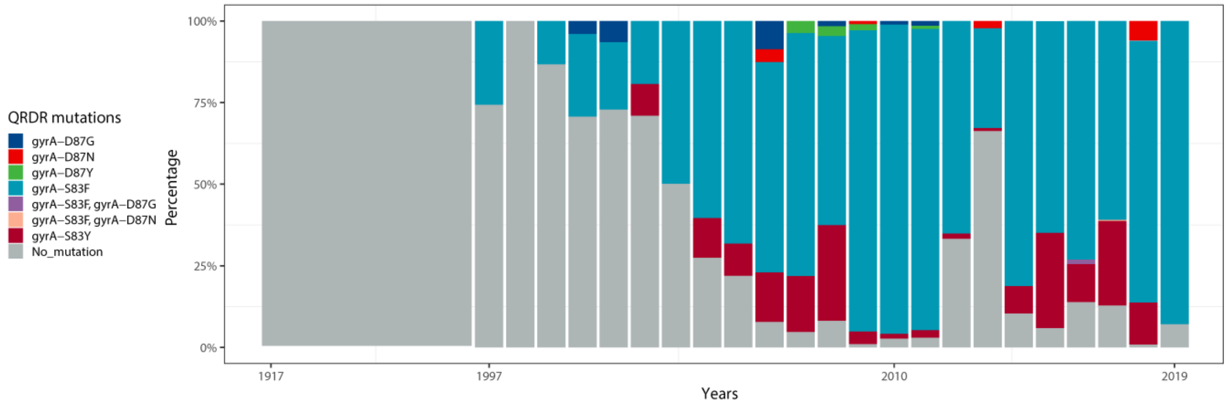

Supplementary Figure 4: QRDR mutations among global *Salmonella* Paratyphi A isolated between 1917 and 2019. A rise in proportion of isolates with QRDR mutations has increased since mid-1990s. Since 2010, a large majority of isolates contain at least one QRDR mutation.

Supplementary Tables

Supplementary Table 1: Selection of isolates from the CHRF *Salmonella* Paratyphi A BioBank for whole-genome sequencing.

CHRF Biobank			Representative sample		
**Year of collection**					
**Year**	**n**	**freq**	**Year**	**n**	**freq**
1999	1	0.2%	1999	1	0.3%
2000	1	0.2%	2000	1	0.3%
2001	1	0.2%	2001	1	0.3%
2004	3	0.5%	2004	2	0.6%
2005	67	10.5%	2005	44	12.6%
2006	134	20.9%	2006	42	12.1%
2007	132	20.6%	2007	55	15.8%
2008	161	25.2%	2008	67	19.3%
2009	14	2.2%	2009	13	3.7%
2010	18	2.8%	2010	17	4.9%
2011	22	3.4%	2011	22	6.3%
2012	13	2.0%	2012	13	3.7%
2013	19	3.0%	2013	19	5.5%
2014	14	2.2%	2014	13	3.7%
2015	18	2.8%	2015	17	4.9%
2016	22	3.4%	2016	21	6.0%
**Patient gender**					
**Gender**	**n**	**freq**	**Gender**	**n**	**freq**
Female	241	38%	Female	135	39%
Male	332	52%	Male	183	53%
NA	67	10%	NA	30	9%
**Collection Sites**					
**Sites**	**n**	**freq**	**Sites**	**n**	**freq**
CMCH	1	0.2%	CMCH	1	0.3%
DMCH	2	0.3%	DMCH	0	0.0%
DSH	177	27.7%	DSH	166	47.7%
KWMCH	2	0.3%	KWMCH	2	0.6%
Popular	360	56.3%	Popular	111	31.9%
SSF	64	10.0%	SSF	60	17.2%
SSMCH	4	0.6%	SSMCH	0	0.0%
NA	30	4.7%	NA	8	2.3%
**Hospital settings**					
**Settings**	**n**	**freq**	**Settings**	**n**	**freq**
IPD	179	28.0%	IPD	163	46.8%
OPD	461	72.0%	OPD	185	53.2%

Supplementary Table 2: Summary of 1,379 isolates used in the study.

Source	N	Freq	Country	Travel data
This study	348	25.1	Bangladesh	NA
SEAP study	469	34.0	Bangladesh, Nepal, Pakistan	NA
Day et al JAC 2018 [1] and Ashton et al PeerJ 2016 [2]	254	18.4	UK (Travel)	Yes (164)
Zhou et al PNAS 2014 [3]	131	9.5	Global	NA
Britto et al PLoS NTD 2018 [4]	66	4.8	Nepal	NA
Kuijpers et al Microb Genom 2016 [5]	54	3.9	Cambodia	NA
Sherchan et al ASTMH 2017 [6]	23	1.7	Nepal	NA
Britto et al JAC 2020 [7]	14	1	Nepal	NA
Yan et al. PLoS NTD 2015 [8]	13	0.9	China	NA
Hooda et al PLoS NTD 2019 [9]	4	0.3	Bangladesh	NA
Nair et al PLoS One 2020 [10]	1	0.07	UK (Travel)	Yes (1)
Holt et al BMC Genomics 2009 [11]	1	0.07	Pakistan	NA
McLelland Nature Genetics 2004 [12]	1	0.07	NA	NA
**Total**	**1379**	**100**		

Supplementary Table 3: List of alleles for the 18 genotypes.

Genotype	Genomic_Location	Allele	Genetic Locus	Gene name, protein
0.1	553085	T	SSPA_RS02400	*aroC,* chorismite synthase
1.0	2073394	T	SSPA_RS10025	cydX, cytochrome bd-I oxidase
1.1	467038	C	SSPA_RS01965	*tkt*, transketolase
1.2	1174035	G	SSPA_RS05540	*narG*, Nitrate reductase subunit alpha
2.0	3387520	G	SSPA_RS16630	*rpsM*, 30S ribosomal protein S13
2.1	1576511	G	SSPA_RS07520	*pheT*, phenylalanine-tRNA ligase subunit beta
2.2	2372711	A	SSPA_RS11445	*cyoB*, cytochrome o ubiquinol oxidase subunit I
2.3	3386591	T	SSPA_RS16620	*rpsD*, 30S ribosomal subunit S4
2.4	865636	T	SSPA_RS03865	*rfbG*, CDP-glucose 4,6-dehydratase
1.2.1	332636	T	SSPA_RS01480	tadA, tRNA adenosine deaminase
1.2.2	4019735	G	SSPA_RS19625	*fdnG*, formate dehydrogenase-N subunit alpha
2.0.1	2082678	G	SSPA_RS10060	*sucA*, 2-oxoglutarate dehydrogenase E1 component
2.3.1	3437895	A	SSPA_RS16940	*trpS*, tryptophan-tRNA ligase
2.3.2	103259	T	SSPA_RS00455	*rsmA*, 16S rRNA adenine demethyltransferase
2.3.3	2419603	G	SSPA_RS11685	*sbcC*, exonuclease subunit
2.4.1	1881695	A	SSPA_RS09100	*rpsA*, 30S ribosomal subunit S1
2.4.2	861876	A	SSPA_RS03840	*rfbD*, dTDP-4-dehydrorhamnose reductase
2.4.3	320372	A	SSPA_RS01410	*lepB*, signal peptidase I
2.4.4	1178060	T	SSPA_RS05545	*narH*, nitrate reductase subunit beta

Note: there are 19 rows for 18 genotypes, as 1.2 was divided into 1.2.1 and 1.2.2; 1.2 is not considered a unique genotype in the current version of Paratype scheme.

Supplementary Table 4: Comparison of run time required by different run modes of the Paratype tool. Genome coverage is also added here to compare with run time. Run time was also calculated for a hypothetical genome of 100x coverage (based on the average run time in comparison to their genome coverage).

ID	SampleID	Genotype	Illumina (fastq mode)		Illumina (bam mode)	Illumina (fasta mode) *	Nanopore (nano mode)	
			Genome coverage	Run time (seconds)	Run time (seconds)	Run time (seconds)	Genome coverage	Run time (seconds)
E119	ERS8226991	1.1	362.0	768.6	15.2	3.5	84.1	149.2
E154	ERS8227025	2.3.2	295.9	600.8	18.8	3.6	54.9	120.2
E205	ERS8227076	2.4.4	179.8	421.4	11.8	3.9	116.7	293.8
E263	ERS8227134	2.4.4	289.9	626.8	17.7	4.2	97.3	245.7
E284	ERS8227155	1.2.2	189.1	434.4	12.6	3.7	110.6	234.7
E285	ERS8227156	2.4.1	243.1	495.9	15.1	4.6	85.1	177.3
E344	ERS8227215	1.2.1	199.2	424.1	14.1	3.8	92.5	188.4
E361	ERS8227232	1.1	254.1	596.2	16.9	3.7	108.3	207.8
E43	ERS8226917	2.3.2	309.2	658.8	20.2	4.1	85.7	199.1
E50	ERS8226924	2.4.1	311.6	656.8	18.6	4.7	131.7	263.5
E81	ERS8226954	1.2.1	344.5	691.6	20.4	3.8	94.1	199.8
**Average run time**		NA	**100**	**214.05[#]**	**16.49**	**3.96**	**100**	**214.8[#]**

All sequencing reads were run on a laptop with 16 threads and 16 GB RAM (12 threads were used). Run time was calculated using time command on Linux.

[*]All Illumina reads were assembled using Unicycler v0.4.9.

[#]Hypothetically calculated for a genome with 100x coverage

Supplementary Table 5: Comparison of *Paratype* results using raw fastq data from Illumina and Nanopore platforms.

ID	Illumina (fastq mode)			Nanopore (nano mode)							Match	Remark
	Run accession	Genome coverage	Genotype	Run accession	Genome coverage	Primary clade	Secondary clade	Subclade	Genotype	Support		
E43	ERR7222171	349.97	2.3.2	ERR9839979	85.68	2	2.3	2.3.2	2.3.2	1	Yes	
E50	ERR7222179	344.58	2.4.1	ERR9837052	131.67	2	2.4	missing	2.4	1	No	Ambiguous ALT nucleotide at 2.4.1 allele position in nanopore data
E81	ERR7222394	376.11	1.2.1	ERR9837053	94.06	1	1.2	1.2.1	1.2.1	0.99	Yes	
E119	ERR7213382	405.19	1.1	ERR9837010	84.11	1	1.1	NA	1.1	0.99	Yes	
E131	ERR7213563	416.91	2.3.3	ERR10170391	37.77	2	2.3	2.3.3	2.3.3	1	Yes	
E134	ERR7213566	459.51	1.2.1	ERR10286194	116.38	1	1.2	1.2.1	1.2.1	0.99	Yes	
E153	ERR7216962	321.01	2.3.2	ERR10170400	23.47	2	2.3	2.3.2	2.3.2	1	Yes	
E154	ERR7216970	324.94	2.3.2	ERR9837018	54.89	2	2.3	2.3.2	2.3.2	1	Yes	
E166	ERR7222486	250.68	2.3.2	ERR10170425	76.75	2	2.3	2.3.2	2.3.2	0.99	Yes	
E205	ERR7220009	201.34	2.4.4	ERR9837020	116.69	2	2.4	2.4.4	2.4.4	0.98	Yes	
E239	ERR7220073	270.49	2.4.4	ERR10170427	22.87	2	2.4	2.4.4	2.4.4	0.99	Yes	
E263	ERR7220098	323.46	2.4.4	ERR9851265	97.28	2	2.4	2.4.4	2.4.4	0.99	Yes	
E268	ERR7220101	252.58	1.2.2	ERR10170428	94.00	1	1.2	1.2.2	1.2.2	1	Yes	
E284	ERR7220124	211.45	1.2.2	ERR9837045	110.62	1	1.2	1.2.2	1.2.2	0.99	Yes	
E285	ERR7222495	268.22	2.4.1	ERR9855449	85.06	2	2.4	2.4.1	2.4.1	1	Yes	
E290	ERR7222496	332.09	1.1	ERR10170431	43.32	1	1.1	missing	1.1	0.95	Yes	
E311	ERR7222499	434.21	2.4.1	ERR10170434	54.35	2	2.4	missing	2.4	0.99	No	Ambiguous ALT nucleotide at 2.4.1 allele position in nanopore data
E342	ERR7220408	361.24	2.4.4	ERR10170481	151.63	2	2.4	2.4.4	2.4.4	1	Yes	

E344	ERR7220410	226.14	1.2.1	ERR9837047	92.51	1	1.2	1.2.1	1.2.1	0.97	Yes	
E361	ERR7222158	299.03	1.1	ERR9837049	108.34	1	1.1	NA	1.1	0.93	Yes	
SEAP7506606	ERR4303933	62.06	2.3.3	ERR10178618	66.01	2	2.3	2.3.3	2.3.3	1	Yes	
SEAP7506645	ERR4304075	85.44	2.4.4	ERR10286193	118.27	2	2.4	2.4.4	2.4.4	0.99	Yes	
SEAP7506707	ERR4362534	73.76	2.3.2	ERR10175979	56.90	2	2.3	2.3.2	2.3.2	1	Yes	
SEAP7506724	ERR4362599	78.15	2.4.1	ERR10286195	71.78	2	2.4	missing	2.4	0.99	No	No ALT nucleotide was found at 2.4.1 allele position in nanopore data
SEAP7506738	ERR4362655	75.35	2.3.2	ERR10175981	37.27	2	2.3	2.3.2	2.3.2	0.97	Yes	
SEAP7506763	ERR4362356	62.41	2.3.3	ERR10175992	40.80	2	2.3	2.3.3	2.3.3	1	Yes	
SEAP7506770	ERR4362537	73.25	1.1	ERR10178617	37.62	1	1.1	missing	1.1	0.96	Yes	
SEAP7506789	ERR4362458	80.81	2.4	ERR10175994	110.33	2	2.4	missing	2.4	0.99	Yes	
SEAP7506811	ERR4362543	73.95	2.4.1	ERR10175995	133.64	2	2.4	2.4.1	2.4.1	0.99	Yes	
SEAP7506820	ERR4362572	77.28	2.3.3	ERR10175996	114.14	2	2.3	2.3.3	2.3.3	1	Yes	
SEAP7506866	ERR4362333	61.49	2.4.1	ERR10176080	114.43	2	2.4	2.4.1	2.4.1	1	Yes	
SEAP7506905	ERR4362479	72.89	2.3.3	ERR10176082	99.41	2	2.3	2.3.3	2.3.3	1	Yes	
SEAP7506936	ERR4362610	67.26	1.1	ERR10176114	107.79	1	1.1	missing	1.1	0.92	Yes	

Supplementary references

1. Day, M. R. *et al.* Comparison of phenotypic and WGS-derived antimicrobial resistance profiles of *Salmonella enterica* serovars Typhi and Paratyphi. *Journal of Antimicrobial Chemotherapy* **73**, 365–372 (2018).
2. Ashton, P. M. *et al.* Identification of *Salmonella* for public health surveillance using whole genome sequencing. *PeerJ* **4**, e1752 (2016).
3. Zhou, Z. *et al.* Transient Darwinian selection in *Salmonella enterica* serovar Paratyphi A during 450 years of global spread of enteric fever. *Proc Natl Acad Sci U S A* **111**, 12199–12204 (2014).
4. Britto, C. D. *et al.* Laboratory and molecular surveillance of paediatric typhoidal *Salmonella* in Nepal: Antimicrobial resistance and implications for vaccine policy. *PLOS Neglected Tropical Diseases* **12**, e0006408 (2018).
5. Kuijpers, L. M. F. *et al.* Genomic analysis of *Salmonella enterica* serotype Paratyphi A during an outbreak in Cambodia, 2013–2015. *Microb Genom* **2**, (2016).
6. Sherchan, J. B. *et al.* Molecular and Clinical Epidemiology of *Salmonella* Paratyphi A Isolated from Patients with Bacteremia in Nepal. *The American Journal of Tropical Medicine and Hygiene* **97**, 1706–1709 (2017).
7. Britto, C. D. *et al.* Persistent circulation of a fluoroquinolone-resistant *Salmonella enterica* Typhi clone in the Indian subcontinent. *Journal of Antimicrobial Chemotherapy* **75**, 337–341 (2020).
8. Yan, M. *et al.* A Large-Scale Community-Based Outbreak of Paratyphoid Fever Caused by Hospital-Derived Transmission in Southern China. *PLOS Neglected Tropical Diseases*

9, e0003859 (2015).

9. Hooda, Y. *et al.* Molecular mechanism of azithromycin resistance among typhoidal *Salmonella* stains in Bangladesh identified through passive pediatric surveillance. *PLOS Neglected Tropical Diseases* **13**, e0007868 (2019).
10. Nair, S. *et al.* Genomic surveillance detects *Salmonella enterica* serovar Paratyphi A harbouring *blaCTX-M-15* from a traveller returning from Bangladesh. *PLOS ONE* **15**, e0228250 (2020).
11. Holt, K. E. *et al.* Pseudogene accumulation in the evolutionary histories of *Salmonella enterica* serovars Paratyphi A and Typhi. *BMC Genomics* **10**, 36 (2009).
12. McClelland, M. *et al.* Comparison of genome degradation in Paratyphi A and Typhi, human-restricted serovars of *Salmonella enterica* that cause typhoid. *Nature Genetics* **36**, 1268–1274 (2004).
