# Supplementary material for: Paratype: a genotyping tool for Salmonella Paratyphi A reveals its global genomic diversity
Source: Nat Commun. 2022 Dec 23;13:7912. doi: 10.1038/s41467-022-35587-6 (PMC9782287; doi:10.1038/s41467-022-35587-6)
Supplement: Supplementary file 2 — Description of Additional Supplementary Files [file 41467_2022_35587_MOESM2_ESM.pdf]

Description of Additional Supplementary Files

Supplementary Data 1 | The accessions of all 1,379 Salmonella Paratyphi A strains included in this study, with metadata and antimicrobial susceptibility data of the 528 strains that were sequenced from Bangladesh.
