# Supplementary material for: Paratype: a genotyping tool for Salmonella Paratyphi A reveals its global genomic diversity
Source: Nat Commun. 2022 Dec 23;13:7912. doi: 10.1038/s41467-022-35587-6 (PMC9782287; doi:10.1038/s41467-022-35587-6)
Supplement: Supplementary file 4 — Reporting Summary [file 41467_2022_35587_MOESM4_ESM.pdf]

nature portfolio

Corresponding author(s): Senjuti Saha

Last updated by author(s): Nov 28, 2022

Reporting Summary

Nature Portfolio wishes to improve the reproducibility of the work that we publish. This form provides structure for consistency and transparency in reporting. For further information on Nature Portfolio policies, see our Editorial Policies and the Editorial Policy Checklist.

Statistics

For all statistical analyses, confirm that the following items are present in the figure legend, table legend, main text, or Methods section.

n/a	Confirmed	
☐	☒	The exact sample size (*n*) for each experimental group/condition, given as a discrete number and unit of measurement
☒	☐	A statement on whether measurements were taken from distinct samples or whether the same sample was measured repeatedly
☒	☐	The statistical test(s) used AND whether they are one- or two-sided<br>*Only common tests should be described solely by name; describe more complex techniques in the Methods section.*
☒	☐	A description of all covariates tested
☒	☐	A description of any assumptions or corrections, such as tests of normality and adjustment for multiple comparisons
☐	☒	A full description of the statistical parameters including central tendency (e.g. means) or other basic estimates (e.g. regression coefficient) AND variation (e.g. standard deviation) or associated estimates of uncertainty (e.g. confidence intervals)
☒	☐	For null hypothesis testing, the test statistic (e.g. *F*, *t*, *r*) with confidence intervals, effect sizes, degrees of freedom and *P* value noted<br>*Give P values as exact values whenever suitable.*
☐	☒	For Bayesian analysis, information on the choice of priors and Markov chain Monte Carlo settings
☒	☐	For hierarchical and complex designs, identification of the appropriate level for tests and full reporting of outcomes
☒	☐	Estimates of effect sizes (e.g. Cohen's *d*, Pearson's *r*), indicating how they were calculated

Our web collection on statistics for biologists contains articles on many of the points above.

Software and code

Policy information about availability of computer code

Data collection	Data collection was done manually, no software was used for data collection.
Data analysis	The following software were used in the analysis: FastQC v0.11.5, Trimmomatic v0.36, Unicycler v0.4.8, Prokka 1.14.5, Roary v3.3, Bowtie2 v2.3.5.l, SAMtools vl.10, BCFtools vl.10.2, Gubbins v2.3.4, RAxML v8.2.12, iTol vS.5, BEAST v1.10.4, TreeAnnotator vl.10, Tracer vl.7.1, FigTree vl.4.4, fastBAPS, PlasmidFinder v2.l, ResFinder v3.2, R v4.0.4, R packages (ggplot2_3.3.3, readxl_1.3.1, dplyr_1.0.6, maps_3.3.0, scatterpie_0.1.6, viridis_0.6.1).<br><br>Custom codes used in this study are available at https://github.com/CHRF-Genomics/Paratype and https://github.com/CHRF-Genomics/CHRF_Paratyphi_scripts.

For manuscripts utilizing custom algorithms or software that are central to the research but not yet described in published literature, software must be made available to editors and reviewers. We strongly encourage code deposition in a community repository (e.g. GitHub). See the Nature Portfolio guidelines for submitting code & software for further information.

Data

Policy information about availability of data

All manuscripts must include a data availability statement. This statement should provide the following information, where applicable:

- Accession codes, unique identifiers, or web links for publicly available datasets
- A description of any restrictions on data availability
- For clinical datasets or third party data, please ensure that the statement adheres to our policy

The raw reads (both Illumina and ONT) of 528 Salmonella Paratyphi A isolates from CHRF, Bangladesh supporting the conclusions of this article are available in the European Nucleotide Archive (ENA) under study accession ERP132884 (n = 348) and ERP112783 (n = 180; from SEAP). The assembled contigs for the genomes are also available under the study accession ERP132884. Raw reads of the isolates from the SEAP project in Nepal and Pakistan are also available on ENA under study accession ERP112783. All accessions are included in Supplementary Data 1. The metadata and antimicrobial susceptibility data supporting the conclusions of this article are also is included in Supplementary Data 1. Source data for the figures (including supplementary figures) are provided with this paper.
All correspondence and material requests should be addressed to Dr. Senjuti Saha (senjutisaha@chrfbd.org)

Human research participants

Policy information about studies involving human research participants and Sex and Gender in Research.

Reporting on sex and gender	The sex distribution of overall Salmonella Paratyphi A positive cases was 241 females and 332 males. For sequencing, samples from 135 females and 183 males was taken, keeping the proportion same as seen in the patient population.
Population characteristics	Child Health Research Foundation in Bangladesh has been preserving invasive Salmonella isolates since 1999 and maintains a biobank of >9000 typhoidal Salmonella isolates, largely from children (<18years of age) that were isolated from the blood of the patients in two different settings: in-patient (hospitalized), and out-patient (community) facility. From this biobank, among 640 Salmonella Paratyphi A isolates collected till December 2016, 348 were randomly selected for whole-genome sequencing (WGS) considering the year of isolation, gender, collection sites, and hospitalization settings (Supplementary Table 1). A set of 469 Salmonella Paratyphi A isolates were also added to this collection, isolated under the Surveillance for Enteric Fever in Asia (SEAP) project from three different typhoid-endemic countries, Bangladesh (n = 180), Nepal (n = 156), and Pakistan (n = 133).
Recruitment	This study included data from 3 sites in Dhaka, Bangladesh: (1) Dhaka Shishu Hospital (DSH), (2) Shishu Shasthya Foundation Hospital (SSFH), and (3) an outpatient-based diagnostic center, the Popular Diagnostic Center (PDC). Patient information, age and sex, was collected if they tested positive for Salmonella Paratyphi A after standard blood culture and biochemical testing. This was done for all positive cases. All tests were conducted as advised by the treating physician, and the data were collected as a part of passive surveillance. There is no possibility of self selection bias.
Ethics oversight	Ethical approval for the parent studies at CHRF (that includes the newly sequenced isolates) was obtained from the Bangladesh Institute of Child Health Ethical Review Committee. In addition, for the SEAP isolates from Nepal and Pakistan, ethical approvals were taken from Nepal Health Research Council, and Aga Khan University Hospital Ethics Committee and Pakistan National Ethics Committee. For the hospitalized cases, informed written consent and clinical information were taken from adult participants and legal guardians of child participants.

Note that full information on the approval of the study protocol must also be provided in the manuscript.

Field-specific reporting

Please select the one below that is the best fit for your research. If you are not sure, read the appropriate sections before making your selection.

[x] Life sciences [ ] Behavioural & social sciences [ ] Ecological, evolutionary & environmental sciences

For a reference copy of the document with all sections, see nature.com/documents/nr-reporting-summary-flat.pdf

Life sciences study design

All studies must disclose on these points even when the disclosure is negative.

Sample size	In total 1,379 Salmonella Paratyphi A were used in the study. No sample size calculation was performed. This included all available sequences of Salmoenlla Paratyphi A genomes in NCBI, and all genomes sequenced by the SEAP study. Among the 640 Salmonella Paratyphi A isolates collected by CHRF in Bangladesh till December 2016, 348 were randomly selected for whole-genome sequencing (WGS) considering the year of isolation, gender, collection sites, and hospitalization settings. No sample size calculation was performed.<br><br>For the BEAST analysis, 315 genomes were randomly selected from the 1,379 isolates. No sample size calculation was performed. A customized python script was used to randomly select two isolates/year/cluster to represent this global collection of Salmonella Paratyphi A, leading to two independent sample sets of 315 isolates each.

Data exclusions	No data was excluded.
Replication	The BEAST analysis was run twice each on the two independently generated sets of 315 isolates. All replicates were successful and the joint effective sample size was greater than 300 for all. No other replication was done as not deemed necessary for this study.
Randomization	For historical samples, all available sequences were obtained. From Bangladesh, 348 samples were sequenced from 640 samples. The selection was done randomly for whole-genome sequencing (WGS) considering the year of isolation, gender, collection sites, and hospitalization settings A set of 469 Salmonella Paratyphi A isolates were also added to this collection, isolated under the Surveillance for Enteric Fever in Asia (SEAP) project from three different typhoid-endemic countries, Bangladesh (n = 180), Nepal (n = 156), and Pakistan (n = 133). The SEAP-Bangladesh isolates (n = 180) were selected using randomization to represent 483 isolates collected between 2016 and 2018. In contrast, SEAP-Nepal isolates included all pre-SEAP isolates (2014 – 2016) and randomly selected SEAP isolates (2017 – 2019). The SEAP-Pakistan isolates were selected prioritizing the availability of geographic information and susceptibility profile during 2016 – 2018. A customized python script was used to randomly select two isolates/year/cluster to represent the global collection of Salmonella Paratyphi A for the BEAST analysis, leading to two independent sample sets of 315 isolates each. There was no experimental groups in this study.
Blinding	No blinding was performed. This is a bioinformatics tool development study, and there were no experimental groups, and no steps requiring blinding.

Reporting for specific materials, systems and methods

We require information from authors about some types of materials, experimental systems and methods used in many studies. Here, indicate whether each material, system or method listed is relevant to your study. If you are not sure if a list item applies to your research, read the appropriate section before selecting a response.

Materials & experimental systems

n/a	Involved in the study	
☒	☐	Antibodies
☒	☐	Eukaryotic cell lines
☒	☐	Palaeontology and archaeology
☒	☐	Animals and other organisms
☒	☐	Clinical data
☒	☐	Dual use research of concern

Methods

n/a	Involved in the study	
☒	☐	ChIP-seq
☒	☐	Flow cytometry
☒	☐	MRI-based neuroimaging
